# *In Vitro* and *In Vivo* Activity of Omadacycline against Two Biothreat Pathogens, Bacillus anthracis and Yersinia pestis

**DOI:** 10.1128/AAC.02434-16

**Published:** 2017-04-24

**Authors:** Judith Steenbergen, S. Ken Tanaka, Lynda L. Miller, Stephanie A. Halasohoris, Jeremy R. Hershfield

**Affiliations:** a Paratek Pharmaceuticals, King of Prussia, Pennsylvania, USA; b Bacteriology Division, U.S. Army Medical Research Institute of Infectious Diseases, Fort Detrick, Maryland, USA

**Keywords:** anthrax, biothreat, omadacycline

## Abstract

The *in vitro* activity and *in vivo* efficacy of omadacycline (OMC) were evaluated against the causative pathogens of anthrax and plague, Bacillus anthracis and Yersinia pestis, respectively. MICs of OMC were determined by broth microdilution according to CLSI guidelines for 30 isolates each of Y. pestis and B. anthracis. The *in vivo* efficacy of omadacycline was studied at a range of dosages in both a postexposure prophylaxis (PEP) murine model of anthrax and plague as well as in a delayed treatment model of inhalational anthrax. Omadacycline was active *in vitro* against Y. pestis (MIC_90_ of 1 μg/ml) and B. anthracis (MIC_90_ of 0.06 μg/ml). Omadacycline was less active *in vitro* than ciprofloxacin (CIP) against Y. pestis (CIP MIC_90_ of 0.03 μg/ml) but was more potent *in vitro* against B. anthracis (CIP MIC_90_ of 0.12 μg/ml). In the mouse model of infection, the survival curves for all treatment cohorts differed significantly from the vehicle control (*P* = 0.004). The median survival for the vehicle-treated controls was 6 days postchallenge, while all antibiotic-treated mice survived the entire study. Omadacycline treatment with 5, 10, or 20 mg/kg of body weight twice daily for 14 days had significant efficacy over the vehicle control in the treatment of aerosolized B. anthracis. Additionally, for postexposure prophylaxis treatment of mice infected with Y. pestis, the survival curves for omadacycline (40 mg/kg twice daily), ciprofloxacin, and doxycycline cohorts differed significantly from the vehicle control (*P* < 0.0001). Omadacycline is potent and demonstrates efficacy against both B. anthracis and Y. pestis. The well-characterized oral and intravenous pharmacokinetics, safety, and tolerability warrant further assessment of the potential utility of omadacycline in combating these serious biothreat organisms.

## INTRODUCTION

In the past 15 to 20 years, the threat of bioterrorism has increased as a result of increasing political and economic unrest in many parts of the world (1, 2). The Centers for Disease Control and Prevention (CDC) has classified bioterrorism agents into three categories, based on their potential to cause severe disease that results in high rates of mortality and according to how readily these agents can be disseminated in the general population (3). Among the bioterrorism agents that pose the highest threat are Bacillus anthracis and Yersinia pestis, which are the causative pathogens for anthrax and plague, respectively. Current antibiotic treatment options against these category A biothreat pathogens are limited, and the potential for engineered antibiotic resistance is high; thus, new therapeutic options are needed for prophylaxis and treatment of the diseases caused by these pathogens (4–8). Few new oral antibiotics are in development for the treatment of biothreat pathogens, and those older agents that have been approved are facing increasing resistance problems and could face engineered resistance.

As a class, tetracyclines have been used for over 60 years and have proven effective and well tolerated for the treatment of a variety of bacterial infections, including those caused by many of the bacterial pathogens considered to be high-priority biologic threats (plague and anthrax). However, reports of resistance to tetracyclines, including doxycycline, and to fluoroquinolones and beta-lactams have appeared in the literature, and these reports highlight the need for new treatment options for these biothreat agents (4). In addition, recent safety concerns for the fluoroquinolones potentially limits their utility (9).

Omadacycline is a novel aminomethylcycline of the tetracycline family that is designed to overcome mechanisms of resistance to the tetracycline class (10–12). The extensive preclinical and clinical development program for omadacycline is based on its demonstrated potent activity against key pathogens for serious community-acquired infections, including methicillin-resistant Staphylococcus aureus, multidrug-resistant Streptococcus pneumoniae, Gram-negative aerobes, and atypical pathogens, and its lack of cross-resistance to older-generation tetracyclines and other antibiotic classes (13–17). Omadacycline is currently in clinical development for acute bacterial skin and skin structure infection (ABSSSI) and community-acquired bacterial pneumonia (CABP) as oral and intravenous (i.v.) monotherapy. Because of its broad *in vitro* spectrum of activity, clinical profile, and oral bioavailability, omadacycline could be well suited for use in the treatment or postexposure prophylaxis (PEP) of infections of concern in both the biodefense and public health settings. This study evaluated the *in vitro* and *in vivo* activity of omadacycline against B. anthracis and Y. pestis.

(This study was previously presented at the ASM Biodefense and Emerging Diseases Research Meeting, Washington, DC, 25 to 27 February 2013.)

## RESULTS

### *In vitro* findings.

**FIG 1:**
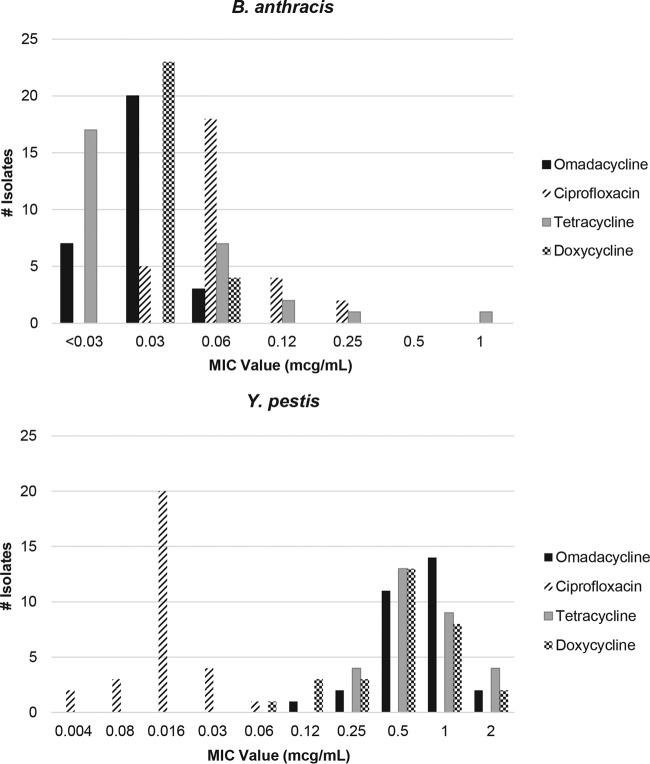
Distribution of MICs (*n* = 30 strains) for omadacycline and comparators for B. anthracis and Y. pestis.

Omadacycline was active against B. anthracis (MIC_90_ of 0.06 μg/ml) and Y. pestis (MIC_90_ of 1 μg/ml). Omadacycline was less potent than ciprofloxacin against Y. pestis (MIC_90_ of 0.03 μg/ml) but slightly more active against B. anthracis (MIC_90_ of 0.12 μg/ml) (Table 1). *In vitro* activity of omadacycline was generally comparable to that of tetracycline and doxycycline. The distribution of MICs for B. anthracis and Y. pestis for omadacycline and comparators is shown in Fig. 1.

**TABLE 1:** MIC values for 30 strains of B. anthracis and Y. pestis for omadacycline and comparators

Strain and parameter	MIC (μg/ml)			
	Omadacycline	Ciprofloxacin	Tetracycline	Doxycycline
B. anthracis (30 strains)				
MIC range	<0.03–0.06	0.03–0.25	<0.03–1	0.03–0.06
MIC_50_	0.03	0.06	<0.03	0.03
MIC_90_	0.06	0.12	0.12	0.06
Y. pestis (30 strains)				
MIC range	0.12–2	0.004–0.06	0.25–2	0.06–2
MIC_50_	1	0.015	0.5	0.5
MIC_90_	1	0.03	2	1

### *In vivo* findings. (i) Study 1.

**FIG 2:**
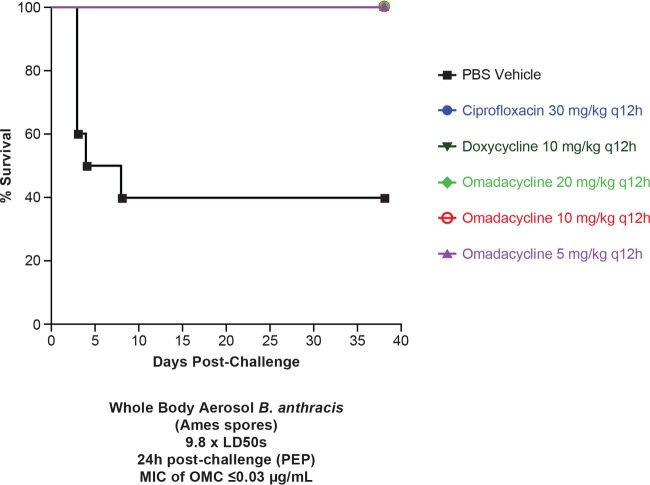
Study 1. Shown is the survival of mice infected with B. anthracis Ames following treatment with omadacycline, doxycycline, or ciprofloxacin (all given i.p.). *n* = 10 for all groups.

The survival curves for all treatment cohorts differed significantly from that of the vehicle cohort (*P* = 0.004) (Fig. 2). The median survival for the vehicle-treated controls was 6 days postchallenge. All omadacycline-treated mice survived the entire study (38 days) regardless of dose studied (5, 10, and 20 mg/kg of body weight twice daily). Additionally, all of the ciprofloxacin (30 mg/kg twice daily)- and doxycycline (10 mg/kg twice daily)-treated mice survived the entire study. Upon study termination, spleens and lungs from surviving mice were excised, and the homogenates were plated on sheep blood agar (SBA) plates to determine the degree of B. anthracis infection. Consistent with this murine model of inhalational anthrax, residual bacteria (mean of ∼6.7 CFU/g for treatment cohorts) were recovered from lungs of each surviving mouse. Spleen culture results for all mice were negative, indicating that surviving mice were cleared of bacterial infection. Positive lung results with negative spleen results are consistent with the infection model (18).

### (ii) Study 2.

**FIG 3:**
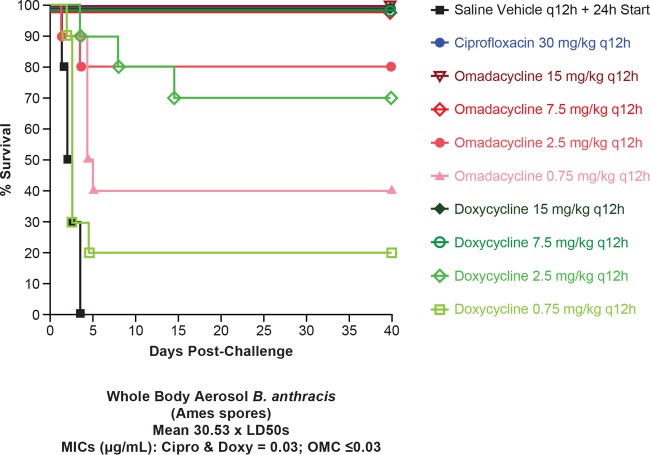
Study 2, postexposure prophylaxis. Shown is the survival of mice infected with B. anthracis Ames following treatment with omadacycline, doxycycline, or ciprofloxacin (all given i.p.). *n* = 10 for all groups (*n* = 9 for ciprofloxacin). The MIC for ciprofloxacin and doxycycline was 0.03 μg/ml, and for omadacycline it was ≤0.03 μg/ml.

In the postexposure prophylaxis arm, the survival curves for the ciprofloxacin, omadacycline (2.5, 7.5, and 15 mg/kg), and doxycycline cohorts differed significantly (*P* < 0.0001) from that of the vehicle cohort (Fig. 3). Furthermore, the doxycycline cohorts receiving 2.5, 7.5, and 15 mg/kg differed significantly (*P* = 0.0354) from each other, but no difference was observed between omadacycline cohorts at the dosages evaluated. The cohort receiving 0.75 mg/kg omadacycline differed significantly (*P* < 0.0004) from the vehicle cohort, but no difference was observed for the matching 0.75-mg/kg doxycycline cohort. Likewise, among the direct comparisons of the four matched omadacycline and doxycycline doses, only the 0.75-mg/kg cohorts differed significantly from each other (*P* = 0.0125). The mean MIC was 0.03 μg/ml for ciprofloxacin and doxycycline and was ≤0.03 μg/ml for omadacycline. All 10 animals died in the vehicle group, with median survival time of 2.25 days. Two animals in the 2.5-mg/kg and 6 animals in the 0.75-mg/kg omadacycline groups died, and median survival time was 4.75 days in the 0.75-mg/kg group.

**FIG 4:**
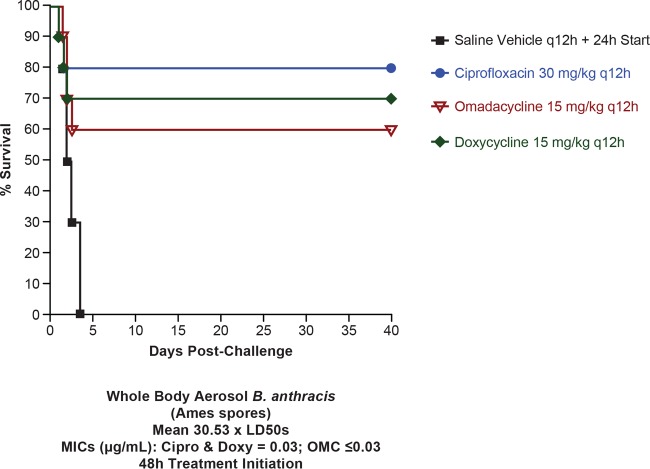
Study 2, delayed treatment (48 h after treatment initiation). Shown is the survival of mice infected with B. anthracis Ames following treatment with omadacycline, doxycycline, or ciprofloxacin (all given i.p.). *n* = 10 for all groups (*n* = 9 for ciprofloxacin). The MIC for ciprofloxacin and doxycycline was 0.03 μg/ml, and for omadacycline it was ≤0.03 μg/ml.

The delayed-treatment mouse efficacy model for anthrax at the United States Army Medical Research Institute of Infectious Diseases (USAMRIID) has been used with both 42- and 48-h postchallenge therapeutic initiation; systemic infection is expected anywhere between 42 and 48 h (18). In this arm of the study, the 30-mg/kg ciprofloxacin cohort differed significantly (*P* = 0.0015) from the vehicle cohort, as did the 15-mg/kg omadacycline cohort (*P* = 0.0177) and the 15-mg/kg doxycycline cohort (*P* = 0.0101) (Fig. 4). No significant differences were observed for delayed treatment among ciprofloxacin, doxycycline, and omadacycline cohorts. The mean MIC was 0.03 μg/ml for ciprofloxacin and doxycycline and was ≤0.03 μg/ml for omadacycline. All 10 animals in the vehicle group died with a median survival time of 2.25 days. Two, four, and three animals in the ciprofloxacin, omadacycline, and doxycycline groups died.

### Y. pestis *in vivo*.

**FIG 5:**
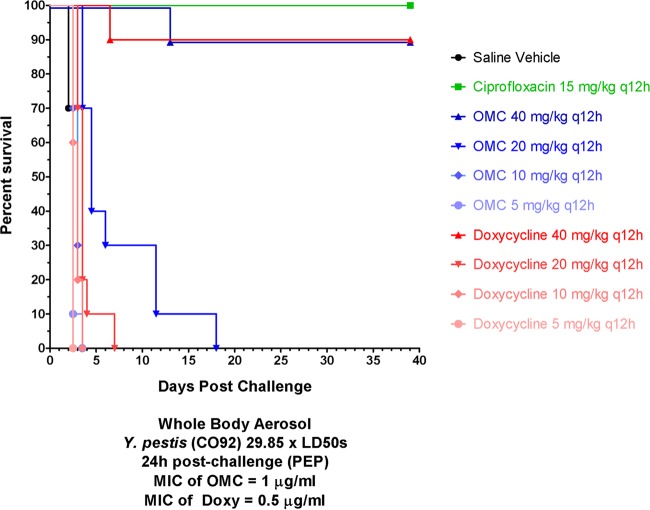
Postexposure prophylaxis. Shown is the survival of mice infected with Y. pestis following treatment with omadacycline, doxycycline, or ciprofloxacin (all given i.p.). *n* = 10 for all groups. The MIC for doxycycline was 0.5 μg/ml, and for omadacycline it was 1 μg/ml.

Ninety percent of omadacycline (40 mg/kg twice daily)- and doxycycline (40 mg/kg twice daily)-treated mice survived the entire study (Fig. 5). All ciprofloxacin-treated mice survived. A dose-response effect for omadacycline and doxycycline was observed, but no significant effect on extended median survival time relative to the saline controls was observed for the 5-, 10-, and 20-mg/kg cohorts. Three representative spleens from each surviving cohort were homogenized and plated for bacteria. No viable bacteria were recovered from all three spleens of the ciprofloxacin and omadacycline (40 mg/kg twice daily) cohorts. In contrast, two of the three doxycycline-treated mice (40 mg/kg twice daily) had Y. pestis cultured from their spleens.

## DISCUSSION

The evaluation of omadacycline demonstrated potent *in vitro* and *in vivo* activity against two category A biothreat pathogens, B. anthracis and Y. pestis. Omadacycline demonstrated broad *in vitro* activity against B. anthracis and Y. pestis isolates, with all isolates having a MIC of ≤2 μg/ml. There was a statistically significant treatment effect of omadacycline, suggesting that omadacycline is an effective postexposure prophylaxis option for treating anthrax, and the lack of mortality in the untreated controls resulted in our studying anthrax in a delayed-treatment exposure model. The data from this model revealed efficacy with omadacycline that was comparable to that of ciprofloxacin and doxycycline. Importantly, the delayed-treatment exposure model is a more robust evaluation of the efficacy of treatments in this model and therefore supports the potential for omadacycline against these pathogens. Understanding the comparative activity of omadacycline to two common agents, doxycycline and ciprofloxacin, is important, as engineered resistance may result in a serious biological threat.

Additionally, results from the higher-inoculum B. anthracis study demonstrated that omadacycline had significant efficacy over the vehicle control in the treatment of aerosolized B. anthracis at doses as low as 0.75 mg/kg daily; in contrast, doxycycline demonstrated a treatment effect at higher doses of 2.5 mg/kg twice daily for 14 days. When dosed sufficiently, both doxycycline and omadacycline were effective as postexposure prophylaxis against inhalational anthrax in this model, and both were comparable to ciprofloxacin.

Similarly, omadacycline, doxycycline, and ciprofloxacin demonstrated significant efficacy against Y. pestis when dosed sufficiently. At 20 mg/kg twice daily, omadacycline significantly outperforms doxycycline but is nonetheless ineffective as PEP against inhalational plague.

While sporadic cases of plague are reported yearly worldwide, a rapid spread of this pathogen as part of a bioterrorism act could have devastating effects on the population. Inhalational anthrax carries with it the most serious complications of biothreat agents and a mortality rate of 90% or more (19–23). While anthrax and plague are generally susceptible to tetracyclines as well as other widely available antibiotic classes, incidents of resistance have been reported, and engineered resistance to these agents may occur.

Based on its *in vitro* activity, its well-characterized pharmacokinetics after oral and i.v. administration, and its safety and tolerability profile, omadacycline offers an attractive treatment option for some of the more serious biothreat organisms evaluated in these studies. As a novel aminomethylcycline compound that has been engineered to overcome existing tetracycline resistance mechanisms of efflux and ribosomal protection, omadacycline might offer a viable treatment alternative where current therapies are not indicated due to host drug reactions or bacterial resistance. This strongly indicates that evaluation in the other infection models should be considered.

## MATERIALS AND METHODS

### *In vitro* study.

MICs were determined by the microdilution method in 96-well plates according to the Clinical and Laboratory Standards Institute (24–26). Antibiotics were serially diluted 2-fold in 50 μl of cation-adjusted Mueller-Hinton broth (CAMHB). The antibiotic ranges were 8 to 0.004 μg/ml or 64 to 0.03 μg/ml based on a final well volume of 100 μl after inoculation.

Bacterial inocula were prepared by suspending colonies into CAMHB from 18 to 24 h (B. anthracis) or 42 to 48 h (Y. pestis) on sheep blood agar (SBA) plates that were incubated at 35°C. Suspended cultures were diluted with CAMHB to a bacterial cell density of 10^5^ CFU/ml adjusted based on the optical density at 600 nm. To each well of the 96-well plate, 50 μl of dilutions was added for a final inoculum of ∼5 × 10^4^ CFU/well. Plates were incubated at 35°C. MICs were determined visually at 18 to 24 h (B. anthracis) or 42 to 48 h (Y. pestis) and also by absorbance at 600 nm (SpectraMax M2; Molecular Devices). Thirty strains, representing the genetic and geographic diversity of each bacterial species, were used in these studies. Quality control of antibiotic stocks was established by using Escherichia coli ATCC 25922, Pseudomonas aeruginosa ATCC 27853, and Staphylococcus aureus ATCC 29213.

The MIC results for tetracycline and doxycycline which were obtained following incubation at 35°C are described elsewhere (27).

### *In vivo* studies. (i) Preparation of B. anthracis and Y. pestis strains.

The United States Army Medical Research Institute of Infectious Diseases (USAMRIID) obtained the B. anthracis Ames strain from the United States Department of Agriculture, Ames, Iowa. It was originally isolated in 1981 from a dead cow in Texas. Purified spore preparations of the organism are maintained at the test facility. B. anthracis Ames spores were prepared according to the method of Leighton and Doi (28). The 50% lethal dose (LD_50_) in mice was 3.4 × 10^4^ CFU inhaled when administered as a whole-body aerosol (18). Spores for aerosol challenge were maintained in sterile water and diluted to the challenge dose of ∼1 × 10^10^ CFU/ml. To verify final bacterial concentrations and exposure doses, following serial dilution and plating at 35°C overnight on sheep blood agar (SBA) plates, colonies were enumerated. The omadacycline and ciprofloxacin MICs against B. anthracis Ames were 0.03 μg/ml.

Y. pestis CO92 was obtained through the NIH Biodefense and Emerging Infections Research Resources Repository and was originally isolated in 1992 from a fatal human case of pneumonic plague. The LD_50_ in mice for this strain was 6.8 × 10^4^ CFU inhaled when administered as a whole-body aerosol. The inoculum for aerosol challenge was prepared as previously described (29), and the suspension of Y. pestis was diluted to the appropriate aerosol challenge dose. To verify final bacterial concentrations and exposure doses, colonies were enumerated after serial dilution and plating on SBA plates incubated for 2 days at 28°C, and colonies were counted. Doxycycline and ciprofloxacin MICs against Y. pestis CO92 were 0.5 μg/ml and 0.06 μg/ml, respectively.

Specific-pathogen-free female BALB/c mice (Charles River Laboratories, Frederick, MD), weighing approximately 20 g, were used throughout the study. Animals were allowed access to food and water *ad libitum* and housed in groups of 10. All procedures were performed in accordance with protocols approved by the USAMRIID Institutional Animal Care and Use Committee and met or exceeded the standards of the American Association for the Accreditation of Laboratory Animal Care (AAALAC), the United States Department of Health and Human Services, and all local and federal animal welfare laws.

### (ii) Aerosol infection.

For study 1, two separate doses of 12.0 and 7.6 times the LD_50_ of B. anthracis were administered. For study 2, four separate doses of 29.8, 27.9, 27.3, and 37.2 times the LD_50_, for a mean of 30.5× LD_50_, were administered. For Y. pestis, mean inhaled doses of 29.4× LD_50_ (3 separate sprays of 26.1×, 32.6×, and 30.8× LD_50_) were administered.

All doses were administered to female BALB/c mice by whole-body aerosol. Challenged mice were then randomized into separate treatment cohorts, balancing challenge doses for each cohort. Aerosols were generated using a three-jet Collison nebulizer (30). All aerosol procedures were controlled and monitored using the automated bioaerosol exposure system (31), operating with a whole-body rodent exposure chamber. Integrated air samples were obtained from the chamber during each exposure using an all-glass impinger (AGI). Aerosol bacterial concentrations were serially diluted and plated on SBA plates as described above. The inhaled dose (CFU/mouse) of B. anthracis or Y. pestis was estimated using mouse respiratory rates according to Guyton (32). Mice were randomly placed into separate cages upon the conclusion of each aerosol.

### (iii) Assessment of efficacy.

For study 1, omadacycline was administered at doses of 5, 10, or 20 mg/kg given twice daily by intraperitoneal (i.p.) injection, beginning 24 ± 1 h after initial B. anthracis challenge. The positive comparator was ciprofloxacin at 30 mg/kg, administered i.p. twice daily for 14 days, starting 24 ± 1 h after challenge. A second comparator group consisting of doxycycline at 10 mg/kg, i.p. twice daily for 14 days, was given 24 h postchallenge to allow comparison of omadacycline to a similar tetracycline class antibiotic. A vehicle control group received 0.1 ml saline i.p. twice daily. Cohorts consisted of 10 animals.

For study 2, cohort size was 10 mice, with the exception of only 9 mice in the postexposure prophylaxis ciprofloxacin group. Omadacycline and doxycycline were administered separately at doses of 0.75, 2.5, 7.5, or 15 mg/kg, given twice daily for 14 days by i.p. injection, beginning 24 ± 1 h after initial B. anthracis challenge (postexposure prophylaxis). Additional omadacycline and doxycycline cohorts received 15 mg/kg twice daily for 14 days beginning 48 ± 1 h after challenge (delayed treatment). The positive comparator was ciprofloxacin at 30 mg/kg, administered i.p. twice daily for 14 days, starting 24 ± 1 h or 48 ± 1 h after challenge. A vehicle control group received 0.2 ml saline i.p. twice daily.

Omadacycline and doxycycline were separately administered at doses of 5, 10, 20 or 40 mg/kg given twice daily for 7 days by i.p. injection, beginning 24 ± 1 h after initial Y. pestis challenge in 10 mice. The positive comparator was ciprofloxacin at 15 mg/kg, administered i.p. twice daily for 7 days, starting 24 ± 1 h after challenge. A vehicle control group received 0.2 ml saline i.p. twice daily.

Survival was assessed at least twice daily during treatment and at least once daily thereafter. Moribund animals were euthanized as necessary and counted as dead. In accordance with the accepted timeline for these animal models of infection, the study was terminated at 38 to 41 days.

### (iv) Drug preparation.

Omadacycline was provided as a tosylate salt (1.38 g of salt form yielded 1 g of active omadacycline). Omadacycline was prepared in batches for 3 days of use and dosed as 0.1-ml i.p. injections (∼20 g/mouse): 20 mg/kg (4 mg/ml) by dissolving 80 mg into 14.5 ml of phosphate-buffered saline (PBS), 10 mg/kg (2 mg/ml) by diluting 4 ml of the 4-mg/ml solution with 4 ml of PBS, and 5 mg/kg (1 mg/ml) by diluting 2 ml of the 4-mg/ml solution with 6 ml of PBS. A commercially supplied 10-mg/ml stock of ciprofloxacin (Teva Pharmaceutical Industries) was diluted to 6.0 mg/ml with sterile water for injection (SWI) for a 0.1-ml injection (∼20 g/mouse) dose of approximately 30 mg/kg i.p. A commercially supplied 100-mg vial of doxycycline (Abraxis BioScience) was resuspended to 10 mg/ml with 10 ml of sterile water for injection and then further diluted with saline to 2 mg/ml for a 0.1-ml injection dose of approximately 10 mg/kg.

### (v) Study analysis.

All analyses were performed by employing a stratified Kaplan-Meier analysis with a log-rank test as implemented in Prism, version 5.04 (GraphPad Software).
